# Supplementary material for: Resolving the full spectrum of human genome variation using Linked-Reads
Source: Genome Res. 2019 Apr;29(4):635–45. doi: 10.1101/gr.234443.118 (PMC6442396; doi:10.1101/gr.234443.118)
Supplement: Supplemental Material [file supp_gr.234443.118_Supplemental_longranger-2.2.zip › longranger-2.2.tar.bz2!/lr2.2-public/lib/stan/cmdstan/stan/doc/api/html/arg__hmc_8hpp_source.html]

Stan: src/stan/gm/arguments/arg\_hmc.hpp Source File


|  |  |
| --- | --- |
|  | Stan  2.5.0  probability, sampling & optimization |


- Main Page
- Related Pages
- Namespaces
- Classes
- Files

- File List
- File Members

All Classes Namespaces Files Functions Variables Typedefs Enumerations Enumerator Friends Macros Pages

- src
- stan
- gm
- arguments

arg\_hmc.hpp

Go to the documentation of this file.

1 #ifndef STAN\_\_GM\_\_ARGUMENTS\_\_HMC\_\_HPP

2 #define STAN\_\_GM\_\_ARGUMENTS\_\_HMC\_\_HPP

3

4 #include <stan/gm/arguments/categorical\_argument.hpp>

5

6 #include <stan/gm/arguments/arg\_engine.hpp>

7 #include <stan/gm/arguments/arg\_metric.hpp>

8 #include <stan/gm/arguments/arg\_stepsize.hpp>

9 #include <stan/gm/arguments/arg\_stepsize\_jitter.hpp>

10

11 namespace stan {

12

13  namespace gm {

14

15  class arg\_hmc: public categorical\_argument {

16

17  public:

18

19  arg\_hmc() {

20

21  \_name = "hmc";

22  \_description = "Hamiltonian Monte Carlo";

23

24  \_subarguments.push\_back(new arg\_engine());

25  \_subarguments.push\_back(new arg\_metric());

26  \_subarguments.push\_back(new arg\_stepsize());

27  \_subarguments.push\_back(new arg\_stepsize\_jitter());

28

29  }

30

31  };

32

33  } // gm

34

35 } // stan

36

37 #endif

38

stan::gm::arg\_hmc

**Definition:** arg\_hmc.hpp:15

arg\_stepsize\_jitter.hpp

categorical\_argument.hpp

stan::gm::categorical\_argument

**Definition:** categorical\_argument.hpp:11

stan::gm::categorical\_argument::\_subarguments

std::vector< argument \* > \_subarguments

**Definition:** categorical\_argument.hpp:153

stan::gm::arg\_engine

**Definition:** arg\_engine.hpp:13

arg\_metric.hpp

arg\_stepsize.hpp

stan::gm::arg\_stepsize

**Definition:** arg\_stepsize.hpp:10

stan::gm::argument::\_name

std::string \_name

**Definition:** argument.hpp:76

arg\_engine.hpp

stan::gm::arg\_hmc::arg\_hmc

arg\_hmc()

**Definition:** arg\_hmc.hpp:19

stan::gm::arg\_stepsize\_jitter

**Definition:** arg\_stepsize\_jitter.hpp:10

stan::gm::argument::\_description

std::string \_description

**Definition:** argument.hpp:77

stan::gm::arg\_metric

**Definition:** arg\_metric.hpp:14


---

[ Stan Home Page ]

*© 2011–2014,
Stan Development Team.*
